# Study on Finite Element Model Updating in Highway Bridge Static Loading Test Using Spatially-Distributed Optical Fiber Sensors

**DOI:** 10.3390/s17071657

**Published:** 2017-07-19

**Authors:** Bitao Wu, Huaxi Lu, Bo Chen, Zhicheng Gao

**Affiliations:** School of Civil Engineering & Architecture, East China Jiao Tong University, Nanchang 330013, China; 2512@ecjtu.jx.cn (H.L.); chenbo4487@163.com (B.C.); 13755692074@163.com (Z.G.)

**Keywords:** model updating, distributed macro-strain, fiber optic sensor, response prediction, bridge

## Abstract

A finite model updating method that combines dynamic-static long-gauge strain responses is proposed for highway bridge static loading tests. For this method, the objective function consisting of static long-gauge stains and the first order modal macro-strain parameter (frequency) is established, wherein the local bending stiffness, density and boundary conditions of the structures are selected as the design variables. The relationship between the macro-strain and local element stiffness was studied first. It is revealed that the macro-strain is inversely proportional to the local stiffness covered by the long-gauge strain sensor. This corresponding relation is important for the modification of the local stiffness based on the macro-strain. The local and global parameters can be simultaneously updated. Then, a series of numerical simulation and experiments were conducted to verify the effectiveness of the proposed method. The results show that the static deformation, macro-strain and macro-strain modal can be predicted well by using the proposed updating model.

## 1. Introduction

With the recent development of sensing monitoring technology, health monitoring and safety assessment for bridges have attracted increasing and extensive attention in civil engineering. Many bridges throughout in the world have been installed with structural health-monitoring systems to monitor the strains, acceleration and frequency and identify the structural performance and damages [1,2,3]. Moreover, the data are also applied to update the Finite Element Model (FEM) of bridges, and then the numerical results agree well with the field-measured ones [4,5,6]. With an updated FE model, the response and performance of a bridge can be predicted well to evaluate its structural health conditions for safe operation [7,8,9].

The main dynamic Finite Element Model Updating (FEMU) method is based on the modal parameters of structures. These modal parameters are divided into two categories: one is based on matrix parameters and the other is based on design parameters. The frequency was selected to construct the objective function based on the sensitivity analysis in early model updating methods. Based on the sensibility analysis of frequency, the parameters with great influence on the sensitivity are selected as the design variables [10,11,12,13]. The application of these methods is based on sensitivity analysis, modal parameter identification and a relatively large amount of computational work, and the updated results may be different due to the selection of different parameters [14]. Kim constructed an objective function that comprised the frequency, displacement modal, and modal strain energy considering the local stiffness, and the damage was only slightly sensitive to the frequency [15]. The dynamic FEMU method based on Frequency Response Function (FRF) has attracted extensive attention. The FRF matrix obtained in vibration tests can be used directly and the errors due to modal analysis can be avoided since the modal analysis is not necessary. The objective function consisting of the error value between the measured FRF and initial model FRF was first established by Lin, and it is minimized the function to update the element stiffness and mass matrices of the FE model [16]. Subsequently, Imregun et al. conducted a series of theoretical studies on the FEMU method based on FRF [17,18,19]. Rahmatalla studied the damage detection from the variation of parameter matrices estimated by incomplete FRF data and the proposed parameter identification method was compared to Kim’s method [20].

Response surface methodology (RSM) is a new method used for bridge FEMU applications in recent years. It combines experimental design and mathematical statistics. Ren studied the RSM based on static and dynamic data, respectively [21,22]. Deng et al. pointed out the shortcomings of this method and developed a new method that used RSM for the best experimental design of the parameters to be updated [23]. There are still some problems when RSM is used for updating bridge structure models. The response surface function of a FE model is fitted out by a certain number of sample result data, and each sample result data requires calculation of a finite model. A large number of finite element calculations are thus required when the number of samples is large.

Many investigations have focused on dynamic FEMU methods, while only a few have considered static FEMU methods. The static measurement data have higher precision and reliability than dynamic test methods such as strain measurement. Both the dynamic and static finite model updating methods are based on the monitoring data of sensors, and accelerometers and strain gauges are commonly used in structural health monitoring. With reference to their spatial disposition, sensors are classified as discrete or point sensors. Discrete strain sensors measure an average strain between two predefined points. The distance between these two points is called the gauge length of the sensor. With respect to the gauge length, the sensors are conventionally classified into two groups: short-gauge and long-gauge sensors. Traditional sensors, such as strain gauges and displacement gauge, belong to the short-gauge sensors group.

An important step in a structural health monitoring strategy is the appropriate selection of the sensor used to measure the required physical variable. Many different types of sensors have been used in SHM, such as acceleration sensors, displacement sensors, cameras, GPS, laser sensors, etc. [24,25].

Traditional short gauge sensors have high precision, but they are more susceptible to other types of measurement error, most notably, errors caused by discontinuities (open cracks) distributed in the monitored material. The measurement information of the traditional point gauge and displacement sensor is too local to reflect the overall characteristics of the structure. The spatially-distributed long-gauge strain sensing technology overcomes the measurement information of the too local traditional point gauge. The global and local information of the bridge can be obtained based on the arrangement of the distributed long-gauge strain sensors throughout the entire structure or critical regions. The availability of long-gauge strain sensors has opened new and interesting possibilities for structural health monitoring. These sensors were proven to be applicable for monitoring at a global structural level through numerous projects [26,27,28,29,30].

Glisic studied the influence of the gauge length on the accuracy of long-gauge sensors employed in monitoring prismatic beams; the relationship between the measurement error and the gauge length was also investigated, and a recommended gauge length was given to ensure that the measurement error is within a reasonable range [31,32].

At present, there is little research on finite element model updating based on long-gauge strain sensors. Hong studied the finite element model updating method based on the macro-strain modal, and the macro strain modal shape was selected as the objective function [33], but the higher order strain modal shapes are not easy to obtain. For complex structures, the influence of higher order strain modes cannot be neglected. In this paper, the macro strain and frequency of the bridge are used as the objective function, and the macro strain mode of the structure is not required, which is convenient to for use in bridge static load tests.

The regular artificial detection and static load tests are used to evaluate the bearing capacity of medium and small span highway bridges. It is necessary to establish the finite element model of the bridge in the static load test, and calculate the equivalent vehicle loads and loading position according to the designed lane loads. The principle of equivalent vehicle load calculation is that the bending moment the key sections of the bridge under the lane loads and the equivalent vehicle loads need to be equal. In this process, the bridge finite element model has not been modified, and the bridge model is established according to the design drawings. After a long period of vehicle load, material aging, corrosion of reinforcement and pre-stress losing, the stiffness of the bridge will degraded over time. It is obvious that the equivalent vehicle load calculated by the initial finite element model will be larger. This is very dangerous for the static and dynamic load tests of old bridges.

Aiming at the above bridge static load experiment problem, we present herein a practical FEMU method that combines dynamic and static long-gauge strain responses for a finite model updating method to modify the finite model in static load tests of highway bridges, which is based on spatially-distributed long-gauge strain sensing. Compared with other finite element methods, the bridge is divided into *N* long-gauge elements, and the element stiffness of the long-gauge elements are selected as the modified parameter. The global and local parameters can be simultaneously modified and only one type of sensor is required in the proposed method. This method combines the advantage of the static and dynamic FEMU methods. A 1:10 scale vehicle and bridge system was established to simulate bridge static load tests. The simulation and experimental results show that the static deformation, macro-strain and the first order macro-strain modal can be predicted well using this updated FE model and the method is suitable for medium-small span bridges with simple structures.

## 2. Objective Function Based on Element Macro-Strain

**Figure 1:**
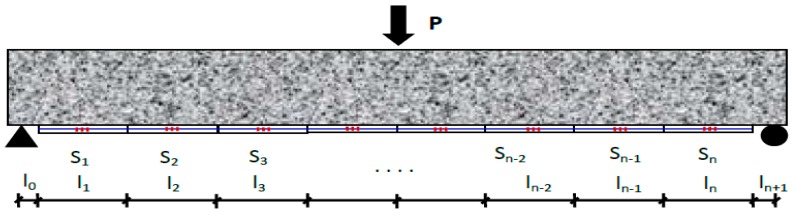
Diagram of the beam.

A simply supported beam was established to study the relationship between the macro-gauge strain and element local stiffness. It is assumed that *N* long-gauge strain sensors with a length of *l_i_* are installed on the bottom of the beam, and named S_1_ to S_n_ from left to right, as shown in Figure 1, which shows the distribution of these long-gauge strain sensors.

The *i-*th macro-strain is solved according to material mechanics and expressed as:

(1) $$\bar{\varepsilon_{i}}=\int_{0}^{l_{i}}\varepsilon dx/l_{i}=\int_{0}^{l_{i}}\frac{\bar{M_{i}}\bar{y_{i}}}{\bar{{\left(EI\right)}_{i}}}dx/l_{i}$$

where $\bar{M_{i}}$, $\bar{y_{i}}$ and $\bar{{\left(EI\right)}_{i}}$ represent the average bending moment, average height of the neutral axis, and average bending stiffness, respectively. Equation (1) is further expressed as:

(2) $$\bar{\varepsilon_{i}}=\int_{0}^{l_{i}}\varepsilon dx/l_{i}=\frac{\bar{y_{i}}}{\bar{{\left(EI\right)}_{i}}l_{i}}\int_{0}^{l_{i}}Mdx$$

Because the average bending moment of sensor S*_i_* (*i* = 1~$\frac{n}{2}$) can be expressed in terms of the force and the distance, the *i-*th macro-strain can be expressed as:

(3) $$\bar{\varepsilon_{i}}=\int_{0}^{l_{i}}\varepsilon dx/l_{i}=\frac{\bar{y_{i}}}{\bar{{\left(EI\right)}_{i}}l_{i}}\int_{0}^{l_{i}}P_{1}(x+\sum_{j=0}^{i-1}l_{j})dx$$

(4) $$\bar{\varepsilon_{i}}=\frac{P_{1}\bar{y_{i}}(\frac{1}{2}l_{i}+\sum_{j=0}^{i-1}l_{j})}{\bar{{\left(EI\right)}_{i}}}\;(i=1\sim \frac{n}{2})$$

where *P*_1_ is the left supporting force, and *P*_2_ is the right supporting force.

Similarly, the average bending moment of sensor S*_i_* (*i* = $\frac{n}{2}+1$~*n*) is expressed as:

(5) $$\bar{\varepsilon_{i}}=\frac{P_{2}\bar{y_{i}}(\frac{1}{2}l_{i}+\sum_{j=i+1}^{n+1}l_{j})}{\bar{{\left(EI\right)}_{i}}}\;\left(i=\frac{n}{2}+1\sim n\right)$$

(6) $$\bar{\varepsilon_{i}}=\frac{R}{\bar{{\left(EI\right)}_{i}}},\;R=\{\begin{array}{cc} P_{1}\bar{y_{i}}(\frac{1}{2}l_{i}+\sum_{j=0}^{i-1}l_{j}) & i=1\sim \frac{n}{2}\\ P_{2}\bar{y_{i}}(\frac{1}{2}l_{i}+\sum_{j=i+1}^{n+1}l_{j}) & i=\frac{n}{2}+1\sim n \end{array}$$

From Equation (5), it can be seen, $P_{2}$, $\bar{y_{i}}$ and $l_{i}$ are the constants, so the *i-*th macro-strain is in inverse proportion to the local average stiffness ${\left(\bar{EI}\right)}_{i}$. This corresponding relation is important significance to verify the local stiffness based on long-gauge strain. The objective function comprising that static macro-strain and frequency was established according to the corresponding relation and it is expressed as follows:

(7) $$f(x)=\sum_{i=1}^{n}\partial_{i}\frac{\left|\varepsilon_{ai}-\varepsilon_{ti}\right|}{\left|\varepsilon_{ti}\right|}+\sum_{j=1}^{m}\beta_{j}{\left(1-f_{aj}/f_{tj}\right)}^{2}$$

in which $\varepsilon_{ai}$ and $f_{aj}$ represent the *i-*th macro-strain and the *j-*th natural frequency from the FE model. $\varepsilon_{ti}$ and $f_{tj}$ represent the *i-*th macro-strain and the *j-*th natural frequency from field measurements, respectively. $\partial_{i}$ and $\beta_{j}$ are weight coefficients. The principle of determination of weight coefficients in this article is $\partial_{1}=1$, and the values of $\beta_{1}$ and $\beta_{2}$ basically that satisfy the macro-strain residual and frequency residual have the same order of magnitude. The macro-strain and low-order modal parameter (frequency) are necessary, which can be easily extracted under dynamic-static load experiment. The FEMU is transferred to minimize the objective function under constrained conditions, and the mathematical model can be written as:

(8) $$\left\{ \begin{array}{l} \min f(x)=\min\{\sum_{i=1}^{n}\partial_{i}\frac{\left|\varepsilon_{ai}-\varepsilon_{ti}\right|}{\left|\varepsilon_{ti}\right|}+\sum_{j=1}^{m}\beta_{j}{\left(1-f_{aj}/f_{tj}\right)}^{2}\}\\ d.v.\;g_{1}\leq g_{i}\leq g_{2}\\ \;h_{1}\leq h_{i}\leq h_{2}\\ \;k_{1}\leq k_{i}\leq k_{2}\\ s.v.\;\varepsilon_{1}\leq \varepsilon_{i}\leq \varepsilon_{2}\\ \;f_{1}\leq f_{i}\leq f_{2} \end{array}\right.$$

where, *g*, *h* and *k* are design variables; g represents the average bending stiffness within the gauge length of a certain long-gauge sensor, and *h* and *k* represent the mass density and boundary condition of the structure, respectively. ε and f are state variables; $\varepsilon_{i}$ represents the macro- strain from the FE model, and $\varepsilon_{1}$ and $\varepsilon_{2}$ are the low and high values of the measurement macro- strain, respectively. $f_{i}$ represents the frequency from the FE model, and $f_{1}$ and $f_{2}$ are the low and high values of the measurement frequency, respectively. The first-order optimization method was applied to solve the mathematical model in this article.

## 3. Numerical Simulation

**Figure 2:**
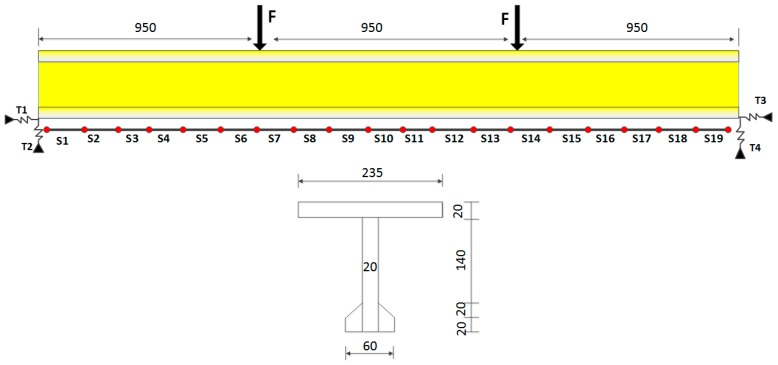
Sensor arrangement and a section of the beam (unit: mm).

A T-shaped beam with 2850 mm span, 235 mm width, and 200 mm height was designed to study the effectiveness of the proposed method (Figure 2). The material properties of the initial FE model of the beam were assigned as follows: the elastic modulus is E = 3.25 × 10^10^ N/m^2^ and the linear mass density is =2500 kg/m^3^, which are measured by experiment. The boundary conditions of the initial FEM are supported by vertical and longitudinal spring element, which are named as T1, T2, T3 and T4 in Figure 2. The initial values of the spring stiffness are T1 = 8.6 × 10^8^ N/m, T2 = 8.79 × 10^8^ N/m, T3 = 8.6 × 10^8^ N/m and T4 = 8.75 × 10^8^ N/m. It is assumed that nineteen macro-strain sensors with a length of 15 cm were installed at the bottom of the beam and named S1–S19 from left to right. The concentrated force that was applied at three dividing points was 5000 N.

**Figure 3:**
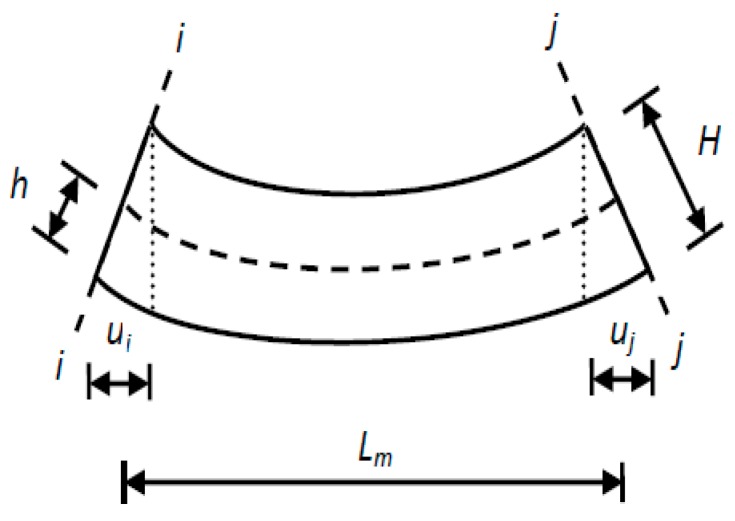
Method for extracting macro-strain.

The *m-*th macro-strain is expressed as:

(9) $${\bar{\varepsilon }}_{m}=\frac{h}{L_{m}}(\nu_{i}-\nu_{j})$$

where, *h* is the distance from the neutral axis to the sensor location and *v*_i_ and *v*_j_ represent the rotational degrees of sections *i* and *j*, respectively (Figure 3). The modal rotation angles *v*_i_ and *v*_j_ can be calculated as follows:

(10) $$\begin{array}{l} \nu_{i}=u_{i}/H\\ \nu_{j}=u_{j}/H \end{array}$$

in which, $u_{i}$ and $u_{j}$ represent the relative displacements of the top and bottom edges of sections *i* and *j*, respectively, and *H* is the height of the beam.

### 3.1. Numerical Analysis Case

**Figure 4:**
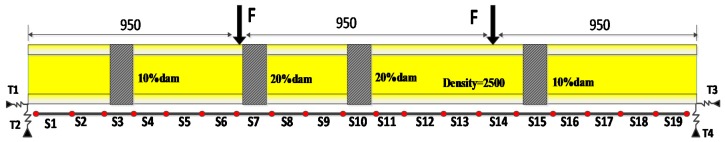
Loaded mode and sensor arrangement in this case (unit: mm).

The local stiffness of the element, the boundary conditions and the mass density were designed as the updated parameters. The actual model with four damages is illustrated in Figure 4. The damages in the case study were simulated with 10%, 20% 20% and 10% reductions in bending stiffness within the gauge length of sensors S3, S7, S10 and S15, respectively. The boundary conditions were changed with a 20% reduction in stiffness of the springs T2 and T4. The mass density was reduced by 20%. The concentrated force applied at three dividing points was 5000 N, and the macro-strain and frequency of the beam in this case were regarded as the experimental values. The initial model was updated based on the experimental values.

### 3.2. Macro-Strain Modal of the Updated Beam

**Figure 5:**
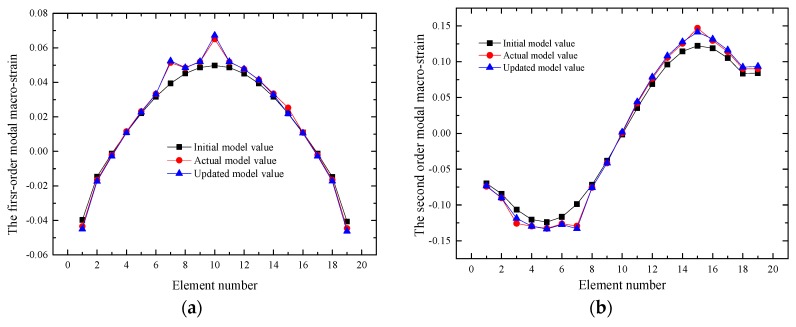
First two-order macro-strain modal curve: (**a**) The first order macro-strain modal curve; (**b**) The second order macro-strain modal curve.

The strain and modal parameters of the long-gauge elements (from S1 to S9) were obtained from the numerical analysis case and the initial FE model was updated based on the distributed macro-strain measurements. The modal macro-strain theory was mainly used to identify the structure damage [27]. Macro-strain modal strain results are very sensitive to local damage, and the first two-order macro-modal strains are extracted from the initial and updated models. The macro-strain modal obtained from the initial and updated models were also compared with the experimental modal macro-strain. Figure 5 shows that the damages can be identified based on the sudden change of the macro-strain modal curves. The results of the case showed that the updated macro-strain modal curve is generally identical to the actual one when local variables, boundary conditions and a global variable (mass density) were modified.

The results of these numerical simulation show that the proposed updating method positively affects the dynamic modification of the local parameters, global parameters and boundary conditions. The damage element E3, E7, E10 and E15 can be identified. The updated model can identify the local damages based on the modal macro-strain.

## 4. Experimental Verification

### 4.1. Experimental Details

#### 4.1.1. Fabrication of Long-Gauge Fiber Bragg Grating (FBG) Sensors and Specimen

**Figure 6:**
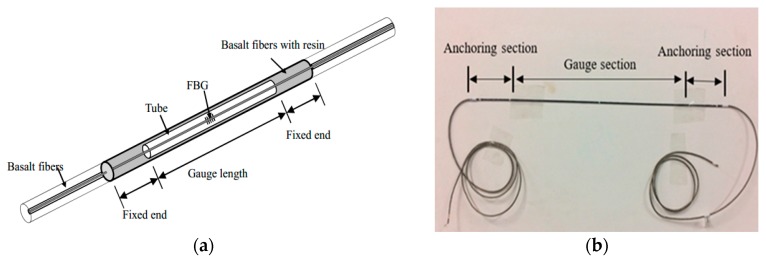
Working principle of long-gauge FBG sensor: (**a**) Diagrammatic sketch of the long-gauge sensor; (**b**) Photo of the long-gauge FBG sensor.

The applied long-gauge strain sensor consisting of two parts—sensing part and connecting optic fiber at two ends—is shown in Figure 6. The sensor is fabricated on a steel calibration instrument. The anchoring section and the gauge section of the sensor are respectively wrapped by a plastic hose with a diameter of 0.9 mm and 1.5 mm, and the plastic hose are packaged in an annular continuous basalt fiber tube (the thermal expansion coefficient of basalt fiber is close to that of concrete).

The middle tube ensures a uniform strain distribution within the gauge length, and therefore the length of the tube is defined as the gauge length of the sensor. The center of the diameter of the sensor casing is far greater than the diameter of the optical fiber, and FBG is in tension state. At the same time, the cured epoxy resin to the sensor provides a certain stiffness, ensure the testing process of the grating sensor in a free state. The long-gauge FBG sensor packaged with basalt fiber reinforced polymer is characterized by a robust protection against harsh environment.

When the sensor is installed on the structure, the average strain of the structure covered by the gauge length can be measured. The gauge length of the long gauge FBG sensors can be designed according to the demand for practical engineering.

**Figure 7:**
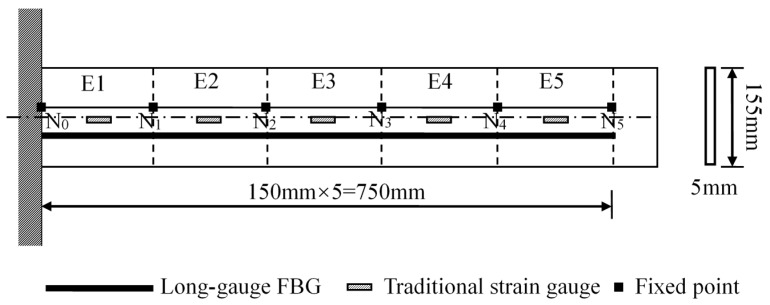
Cantilever sensor layout (top view).

A cantilever dynamic experiment is designed to verify the sensing performance of the long-gauge FBG sensor, and the traditional strain gauges and long-gauge FBG sensors were installed on the bottom. The material of the cantilever beam is steel with 155 mm in height and 5 mm in width. Cantilever beam is divided into five elements named as E1 to E5 from left to right. Sensor arrangement is shown in Figure 7.

**Figure 8:**
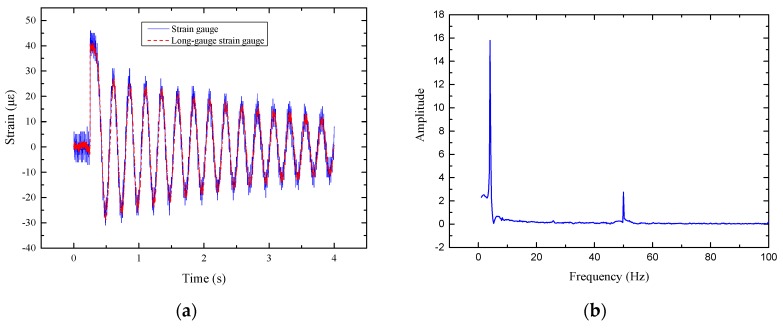
Verification test of long gauge FBG sensor: (**a**) Comparison of the dynamic strain; (**b**) PSD of the dynamic strain.

The dynamic strain of E3 measured by strain gauge and long-gauge FBG sensor are shown in Figure 8a. It can be seen from this figure that the noise of the strain response measured by the strain gauge is larger than that measured by the long-gauge FBG sensor and the long-gauge FBG has high accuracy. PSD analysis of the macro-strain is shown in Figure 8b and the two-order natural frequencies of the structure were obtained.

#### 4.1.2. Experiment Setup

**Figure 9:**
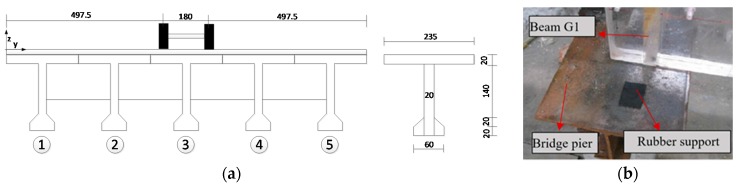
Location of the vehicle and simulation of the rubber support: (**a**) Location of the vehicle (unit: mm); (**b**) Simulation of the rubber support.

A 1:10 scale bridge model is designed and tested according to the highway five T-beam bridges with 30 m in length and 11.75 m in width. Figure 9a schematically shows the section dimensions of the model bridge and the vehicle locations. The model bridge was built with plexiglass. The simply supported model bridge has a span of 3 m, a roadway width of 1.175 m and five girders, which are named as G1, G2, G3, G4 and G5 from left to right, as shown in Figure 9a. The girder height is 0.215 m, and the bridge deck thickness is 0.028 m. The rubber support of the bridge is simulated with a rubber pad (as shown in Figure 9b).

**Figure 10:**
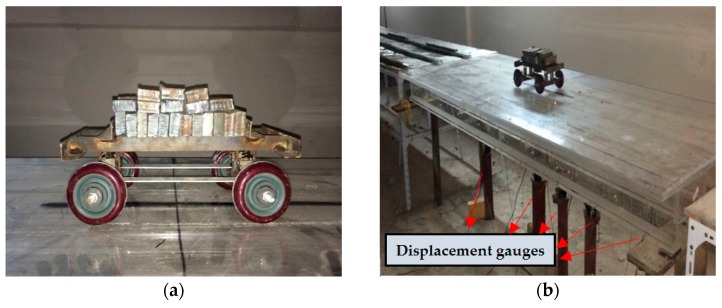
Model vehicle and load application: (**a**) Model vehicle and the mass; (**b**) Vehicle load application.

A model vehicle is designed to simulate the static-loading test on the model bridge (Figure 10a). The weight of the model vehicle is 3.7 kg, and fifty masses were designed, each of which is 0.57 kg. The wheelbase and wheel track of the model vehicle were 0.36 m and 0.18 m, respectively. There are four load grades: load grades 1, 2, 3 and 4 were the weight of the vehicles with 20, 30, 40 and 50 masses, respectively. Load application is shown in Figure 10b.

**Figure 11:**
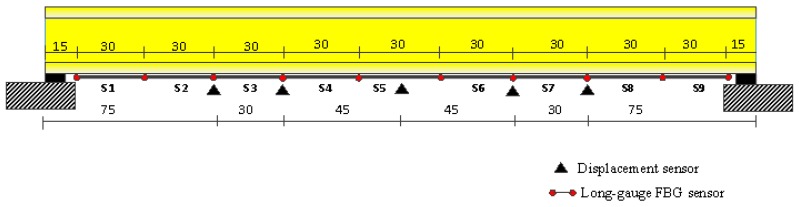
Sensor placement (unit: cm).

**Figure 12:**
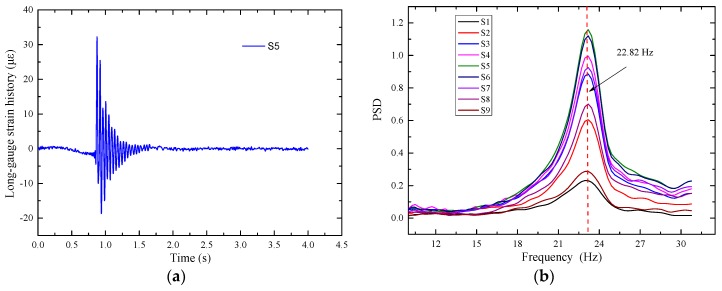
Long-gauge strain history and PSD of the long-gauge strain history: (**a**) Long-gauge strain history of S5; (**b**) PSD of the strains history.

The details of the girder dimensions and sensor placement are shown in Figure 11. On the bottom surface of the T-beams, nine long-gauge strain sensors with a gauge length of 300 mm (S1–S9) are installed. Each T-beam is divided into nine regions and the model bridge is divided into forty-five regions. Five displacement sensors (D1–D5) are also installed on the G3 beam for comparison. Dynamic excitation with arbitrary amplitude using an impulse hammer is successively applied several times at each node of the bridge deck, and the macro-strain response was measured with a sampling rate of 1000 Hz. The long-gauge strain history of S5 on the G3 T-beam is shown in Figure 12a. The analysis of the power spectral density function (PSD) on the long-gauge strain response of the G3 T-beam is shown in Figure 12b, and the first-order frequency of 22.82 Hz is obtained.

### 4.2. Model Updating Based on the Macro-Strain Response

**Figure 13:**
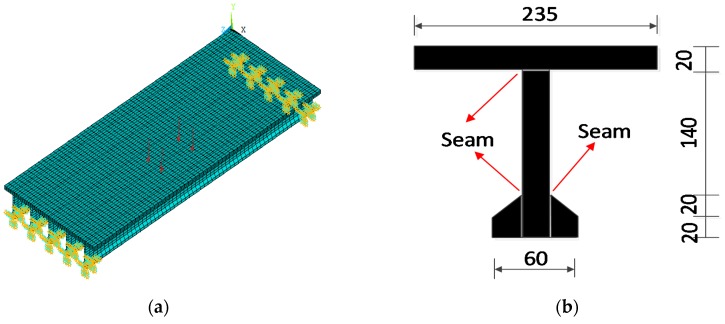
Load applied on FE model and location of the updated elements: (**a**) Load applied on FE model; (**b**) T-beam is composed of two trapezoid and two rectangular sections (Unit: mm).

A FE model of the model bridge was established (Figure 13a). The model is established using the ANSYS software. Three types of elements are used in the FE model, the main girder is built with the solsh190 element, the pavement is modeled with the shell63 element, and the support is simulated by the combin14 element. The bridge material was plexiglass, with an elastic modulus of 3.25 × 10^9^ N/m^2^, a density of 1170 kg/m^3^, and a Poisson ratio of 0.26. Each element was 0.05 m long, and each strain sensor covered six elements. In total, nine long-gauge strain sensors were used for each T-beam. The boundary conditions of the initial FE model were supported by vertical and longitudinal spring elements, which were called T15, T17 (left support), T16 and T18 (right support), as shown in Figure 13. The initial stiffness coefficient of the left support was T15 = 8.19 × 10^5^ N/m (vertical, y-direction) and T17 = 2.75 × 10^2^ N/m (longitudinal, z-direction); the right support springs were T16 = 8.19 × 10^5^ N/m (vertical, y-direction) and T18 = 2.75 × 10^2^ N/m (longitudinal, z-direction). Each support consisted of two row vertical and longitudinal spring elements with a spacing of 10 cm, according to the size of the rubber pads. The initial spring stiffness coefficient is estimated by finite element static calculation according to the static model bridge experiment.

Four concentrated forces are equivalent to the vehicle loads, and these forces were applied on the FEM at the location of the wheels on the model bridge as shown in Figure 13a. The long-gauge strain and modal macro-strain can be solved using Equation (9). The measured macro-strains under load grade 1 were used to update the FE model, and the local stiffness and boundary conditions were the design variables.

According to the sensitivity analysis of parameters in existing methods, the elastic modulus, boundary condition and density of the finite element model have a high sensitivity to the model modification, and therefore are often chosen as the parameters to be modified. In this paper, the density of the material is measured as a constant value. Therefore, the stiffness of the long-gauge element and the boundary condition of the bridge are selected as the modified parameters. Because the element stiffness EI is equal to the product of the elastic modulus and the moment of inertia, the elastic modulus of the element E is chosen as the modified parameter.

The T-beam is composed of two trapezoid and two rectangular sections, which are bonded using chemical glue (Figure 13b). Therefore, the global and local stiffness values were lower than those of the FE model, which was established without considering the stiffness reduction. The stiffness in the black part elements was chosen as the design variables to simulate the stiffness reduction during the production of the model bridge.

**Figure 14:**
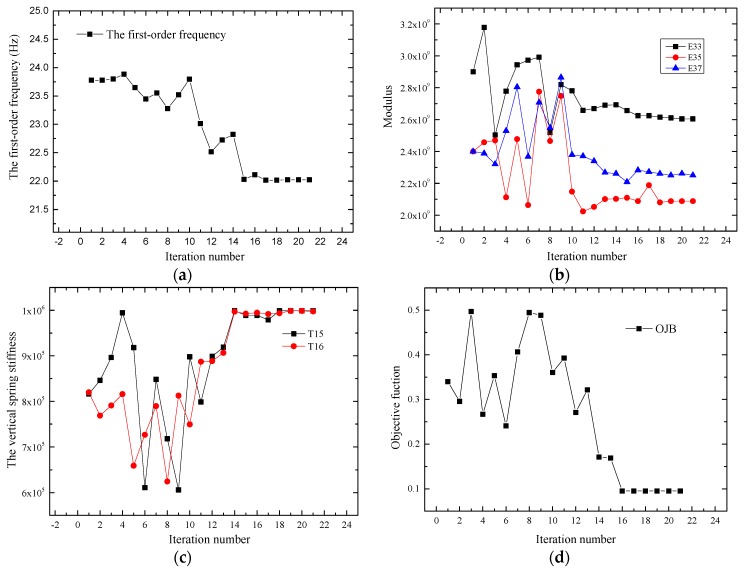
Typical convergence of the calculation process: (**a**) Convergence of the first-order frequency; (**b**) Convergence of the modulus; (**c**) Convergence of the vertical stiffness; (**d**) Convergence of the objective function.

The finite element model of the bridge is updated with the ANSYS software, and the optimization algorithm is the optimal gradient method. The typical convergence of the objective function, bending stiffness and boundary conditions is shown in Figure 14.

The FEMU method based on the distributed long-gauge strains sensor technology easily converges. The frequency of the model stabilized at approximately 22.21 Hz, as illustrated in Figure 14a. The identified local modulus E of the S3, S5 and S7 covered gauges is illustrated in Figure 14b. The modulus of the element increased if the measured strain value was lower than the initial calculated strain, such as E33. E33, E35 and E37 represent the third, fifth and seventh long-gauge element in beam G3, respectively. Similarly, the modulus of the element decreased when the measured strain value was higher than the initial calculated strain. These results verified that the macro-strain was inversely proportional to the local stiffness, which was detected using the long-gauge strain sensor. The convergence of the vertical spring stiffness values is shown in Figure 14c. The objective function converged to approximately 0.1, as shown in Figure 14d.

The updated results of the local elastic modulus are listed in Table 1, and those of the support spring stiffness and frequencies are listed in Table 2. E1–E9 represents the long-gauge elements of each beam in the table. It can be seen from the Table 2; the first order frequency of the updated FE model is more closed to the measured frequency. The parameters of the support are also updated.

### 4.3. Prediction Response Based on the Updated Model

#### 4.3.1. Prediction of the Macro-Stain Response

**Figure 15:**
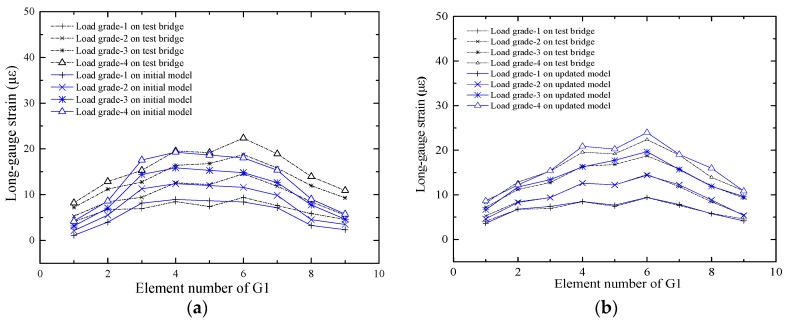
Comparison of macro-strains of the G1: (**a**) Comparison between the initial strain and the actual strain; (**b**) Comparison between the updated strain and the actual strain.

**Figure 16:**
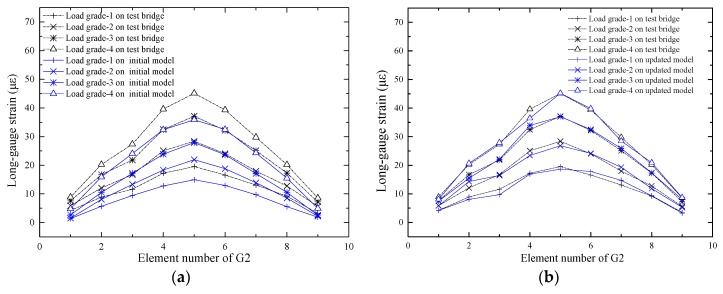
Comparison of long-gauge strains of G2: (**a**) Comparison between the initial strain and the actual strain; (**b**) Comparison between the updated strain and the actual strain.

**Figure 17:**
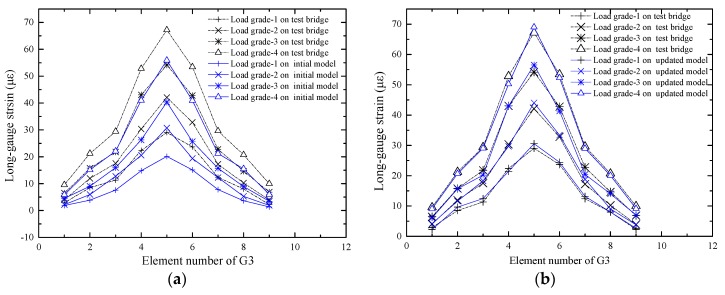
Comparison of macro-strains of the G3: (**a**) Comparison between the initial strain and the actual strain; (**b**) Comparison between the updated strain and the actual strain.

Predicting the response of the structure is one of the purposes of the FEMU technology. To verify the effect of the updating method, the macro-strains of G1, G2 and G3 were extracted from the updated model under load grades 1, 2, 3 and 4. The initial response and the measured macro-strain were compared, as shown in Figure 15a, Figure 16a and Figure 17a.

**Figure 18:**
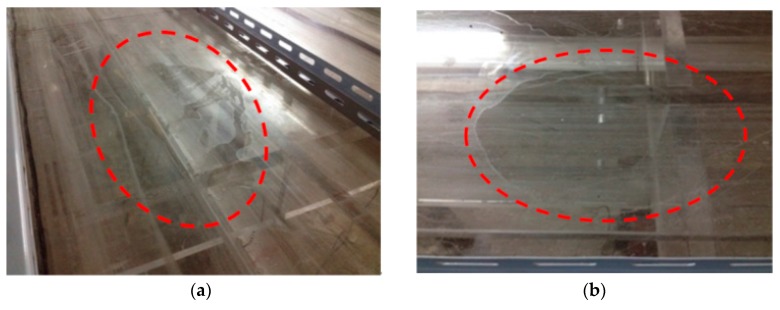
Defect in the model bridge: (**a**) Overall view of the bridge deck; (**b**) Detail view of the defect in the bridge deck.

It is shown in Figure 15a that the difference between the initial value and the measured value is small, which indicates that the decrease in bending stiffness of the G1 beam is small. The difference between the initial value and the measured value of the beam G3 is notably large, as shown in Figure 17a, which indicates that the decrease in bending stiffness of the beam G3 is large. The predicted response and the measured macro-strains are compared as shown in Figure 15b, Figure 16b and Figure 17b, which show that the updated values are notably close to the actual values. Table 1 shows that most elastic modulus values of the long-gauge element decreased because the stiffness decreased during the production of the model bridge. Figure 18 shows that the gap between the bridge deck and the T-beam. The bridge deck is an intact plate and is glued with five T-beams using chemical glue; the T-beam is composed of five small plates and there are three seams in each beam. Therefore, there are many seams in the model bridge, which leads to the degradation of the bridge bending stiffness. There are also many potential defects and visible damage during the construction and operation of the bridge therefore the initial defect and stiffness degradation of the bridge must be considered in the finite element model. When the finite element model is used to evaluate the bearing capacity of the bridge and the accuracy of the finite element model is directly related to the reliability of the evaluation results.

#### 4.3.2. Prediction of the Displacement Deformation

**Figure 19:**
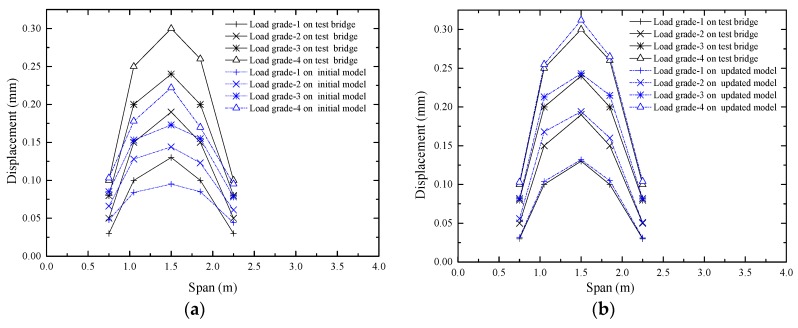
Comparison of the displacement of G3: (**a**) Comparison between the initial displacement and the actual displacement; (**b**) Comparison between the updated displacement and the actual displacement.

The displacements of the beam G3 were measured during the experiment, and the layout of the displacement sensors is shown in Figure 11. The displacements of the beam G3 were extracted from the updated model under load grades 1, 2, 3 and 4. The initial response and the measured displacement were compared, as shown in Figure 19a. The updated response and the measured displacement were compared, as shown in Figure 19b, which indicates that the updated values are notably close to the actual values.

#### 4.3.3. Prediction of the First Order Macro-Strain Modal

**Figure 20:**
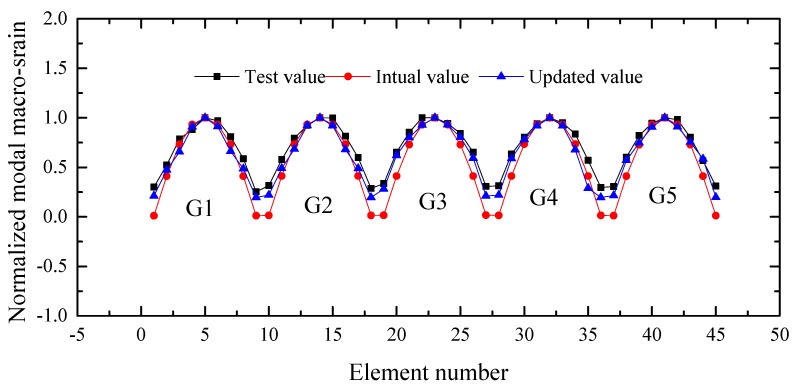
Comparison of the first-order modal macro-strains.

The macro-strain modal is key part of the distributed long-gauge strain sensing in structural health monitoring. The macro-strain modal curve is formed by the peak value of the frequency amplitude, which can be obtained by Fourier transform of the long-gauge strain time histories. The strain modal curve is one of the important modal parameters of the bridge. When there is no damage in the long-gauge element, the macro-strain modal curve is a smooth curve. When the damage occurs in the long-gauge element, there are will be bulges at the location of the damage elements. The first-order macro-strains modal were extracted from the initial and updated modal, and the extraction method is shown in Equation (9). The normalized modal macro-strains that were obtained from the initial and updated models were also compared with the normalized experimental modal macro-strain, as shown in Figure 20.

The result shows that the updated macro-strain modal is notably close to the experimental value. The macro-strain modal of G1, G2, G3, G4 and G5 beam are shown from 1 to 9, 10 to 18, 19 to 27, 28 to 36 and 37 to 45 in the horizontal axis, respectively. It can be found from Figure 20 that the first order macro-strain modal curves of the five T-beams are basically smooth, which shows that the whole stress condition of the bridge is good.

In general, some obvious characteristics of the updating method can be summarized based on the comparisons of the static macro-strain, displacement and macro-strain modal between the numerical simulation and the experiment: (1) the global and local parameters can be simultaneously modified, and the calculation process easily converges; (2) the dynamic and static characteristics can be updated; and (3) only one type of the long-gauge strain sensor is necessary. It should be noted that the modified parameters in this paper are high sensitivity in the finite element updating method, which has been studied in many methods. Therefore, the influence of other parameters on the model updating method need be further studied.

## 5. Conclusions

A practical updating method that combines dynamic and static finite models is presented, which is based on the distributed macro-strain sensor technology. The proposed method is mainly aimed at the problem that the finite element model has not been modified when load test is carried on the highway bridge. It can be concluded as:

1. The macro-strain is inversely proportional to the local average element stiffness that is covered by the gauge. The corresponding relation is important to verify the local stiffness based on the macro-strain. The objective function of the static macro-stains and frequency was established, and the local bending stiffness and boundary conditions of structures can be selected as the design variables.
2. The macro-strain, displacement and first-order macro-strain modal of the updated model are compared with the measured response. The prediction response is notably close to the actual values, and the results show that the dynamic and static characteristics can be updated. The local and global parameters can be simultaneously modified and only one type of sensor is required in the proposed method.
3. The proposed method is mainly useful for medium-small span beam-like bridges in highways. However, it is unrealistic to place long-gauge strain sensors all over the bridge. There is still some work to be done in the future to make the proposed method more useful and applicable. Different types of beam sections and actual bridge models should be investigated to further verify the proposed method, and the optimal sensor placement and number require further research.

## Figures and Tables

**Table 1:** Elastic modulus of the updated elements.

	Initial Value	Updated Value (Pa)								
	E1–E9	E1	E2	E3	E4	E5	E6	E7	E8	E9
G1	3.12 × 10^9^	2.5 × 10^9^	2.6 × 10^9^	2.5 × 10^9^	2.5 × 10^9^	2.4 × 10^9^	2.5 × 10^9^	2. 5 × 10^9^	2. 5 × 10^9^	2.5 × 10^9^
G2	3.12 × 10^9^	2.5 × 10^9^	2.5 × 10^9^	2.4 × 10^9^	2.5 × 10^9^	2.3 × 10^9^	2.3 × 10^9^	2. 3 × 10^9^	2. 3 × 10^9^	2.5 × 10^9^
G3	3.12 × 10^9^	2.4 × 10^9^	2.7 × 10^9^	2.6 × 10^9^	2.5 × 10^9^	2.3 × 10^9^	2.3 × 10^9^	2.3 × 10^9^	2.4 × 10^9^	2.4 × 10^9^
G4	3.12 × 10^9^	2.4 × 10^9^	2.4 × 10^9^	2.4 × 10^9^	2.4 × 10^9^	2.3 × 10^9^	2.4 × 10^9^	2. 4 × 10^9^	2. 4 × 10^9^	2.4 × 10^9^
G5	3.12 × 10^9^	2.5 × 10^9^	2.5 × 10^9^	2.5 × 10^9^	2.4 × 10^9^	2.4 × 10^9^	2.4 × 10^9^	2. 4 × 10^9^	2. 4 × 10^9^	2.4 × 10^9^

**Table 2:** Parameter of the support and the frequency.

	Initial Value	Updated Value	Measured Value
F (Hz)	23.85	22.21	22.82
T15 (N/m)	8.19 × 10^5^	9.98 × 10^5^	/
T16 (N/m)	8.19 × 10^5^	9.97 × 10^5^	/
T17 (N/m)	2.75 × 10^2^	1.58 × 10^2^	/
T18 (N/m)	2.75 × 10^2^	1.37 × 10^2^	/
